# Should we reconsider how to assess eutrophication?

**DOI:** 10.1093/plankt/fbad022

**Published:** 2023-05-25

**Authors:** L Polimene, O Parn, E Garcia-Gorriz, D Macias, A Stips, O Duteil, N Ferreira-Cordeiro, S Miladinova, C Piroddi, N Serpetti

**Affiliations:** European Commission, Joint Research Centre, Directorate D – Sustainable Resources, Via Enrico Fermi 2749, 21027, Ispra (VA), Italy; European Commission, Joint Research Centre, Directorate D – Sustainable Resources, Via Enrico Fermi 2749, 21027, Ispra (VA), Italy; European Commission, Joint Research Centre, Directorate D – Sustainable Resources, Via Enrico Fermi 2749, 21027, Ispra (VA), Italy; European Commission, Joint Research Centre, Directorate D – Sustainable Resources, Via Enrico Fermi 2749, 21027, Ispra (VA), Italy; European Commission, Joint Research Centre, Directorate D – Sustainable Resources, Via Enrico Fermi 2749, 21027, Ispra (VA), Italy; European Commission, Joint Research Centre, Directorate D – Sustainable Resources, Via Enrico Fermi 2749, 21027, Ispra (VA), Italy; European Commission, Joint Research Centre, Directorate D – Sustainable Resources, Via Enrico Fermi 2749, 21027, Ispra (VA), Italy; European Commission, Joint Research Centre, Directorate D – Sustainable Resources, Via Enrico Fermi 2749, 21027, Ispra (VA), Italy; European Commission, Joint Research Centre, Directorate D – Sustainable Resources, Via Enrico Fermi 2749, 21027, Ispra (VA), Italy; European Commission, Joint Research Centre, Directorate D – Sustainable Resources, Via Enrico Fermi 2749, 21027, Ispra (VA), Italy

**Keywords:** eutrophication, eutrophication indexes, trophic interactions, marine biogeochemical models

## Abstract

Eutrophication in marine waters is traditionally assessed by checking if nutrients, algal biomass and oxygen are below/above a given threshold. However, increased biomass, nutrient concentrations and oxygen demand do not lead to undesirable environmental effects if the flow of carbon/energy from primary producers toward high trophic levels is consistently preserved. Consequently, traditional indicators might provide a misleading assessment of the eutrophication risk. To avoid this, we propose to evaluate eutrophication by using a new index based on plankton trophic fluxes instead of biogeochemical concentrations. A preliminary, model-based, assessment suggests that this approach might give a substantially different picture of the eutrophication status of our seas, with potential consequences on marine ecosystem management. Given the difficulties to measure trophic fluxes in the field, the use of numerical simulations is recommended although the uncertainty associated with biogeochemical models inevitably affects the reliability of the index. However, given the effort currently in place to develop refined numerical tools describing the marine environment (Ocean Digital Twins), a reliable, model-based, eutrophication index could be operational in the near future.

## INTRODUCTION

The term eutrophication derives from the ancient Greek words “eu”, which means “well”, and “trope” which means “nourishment”. As such, the word does not intrinsically define something negative. During the years, however, several definitions have been elaborated all involving the concept of ecosystem dysfunctionality induced by an excess of nutrients due to human activities. Negative effects due to increased nutrient loads include an increase in primary production (PP) and biomass, a decrease in light penetration in the water column and a reduced level of oxygen in the bottom layers (Diaz and Rosenberg, 2008). Additionally, nutrient imbalance often associated to large riverine nutrient inputs could lead to the development of harmful algal blooms (Granéli *et al*., 2008). Given the potential damage to ecosystem goods (e.g. seafood) and services (e.g. tourism) these events may induce, eutrophication is generally regarded as one of the main issues to face in order to improve the quality of coastal marine ecosystems (Piroddi *et al*., 2021a). The most important European regulation on marine environment, the Marine Strategy Framework Directive, clearly states that minimizing eutrophication is one of the goals to achieve good environmental status (GES). However, although the impacts of excess nutrients are well known (e.g. Ferreira *et al*., 2011), it is still difficult to objectively establish the limits above which nutrients may trigger undesirable effects (Karydis, 2009) and, in general, the biogeochemical thresholds (nutrients, phytoplankton biomass and dissolved oxygen) to discriminate between areas affected by eutrophication and those not.

Threshold values (TVs) are indeed expected to vary, in space, due to changing environment (distance from the shore, bathymetry, sediment quality and hydrodynamic conditions, Karydis, 2009) and time, due to seasonality and, on longer time scales, climate change (Glibert, 2012). This implies that the same value of a given indicator can have different implications depending on the environmental context. For example, the same concentration of phytoplankton biomass may have different fates and impacts depending on the dominant functional type contributing to that biomass. Edible, highly palatable, phytoplankton species will be efficiently grazed by zooplankton (Mitra, 2006; Polimene *et al*., 2015) with positive effects on high trophic levels and fisheries (Nixon and Buckley, 2002). To the contrary, if inedible or low palatable phytoplankton is dominant, most of the photosynthesized carbon will remain within the low trophic levels of the food chain (Chislock *et al*., 2013), resulting in the production of particulate and dissolved detritus (due to non-predatory losses, Eddy *et al*., 2021), an increase in microbial respiration and reduced oxygen; all dysfunctional events typically associated to eutrophication (EEA, 2019). Similarly, high nutrient concentration may have different and unpredictable impacts depending on the nutritional status of the local microbial population (N- vs. P-limited), as suggested by Krom *et al*. (2005), who observed an increase in heterotrophic activity but not PP in nutrient addition experiments. Such variability in ecosystem responses would imply a continuous redefinition of concentration thresholds that should also consider the evolution of the ecosystem toward new stable states (Glibert, 2012). For example, a threshold for phytoplankton biomass should account for community composition and zooplankton feeding behavior (Sailley *et al*., 2015) and a threshold for nutrients should account for the local environmentalstoichiometry, the presence of organic matter and the competition exerted by bacteria on phytoplankton nutrient acquisition (Krom *et al*., 2005; Polimene *et al*., 2006 and 2017). In other words, eutrophication, like any ecosystem response to pressures, is an emergent property implying a systemic response that is not possible to quantify by assessing single biogeochemical variables, even if combined.

For a more accurate assessment of eutrophication, we should therefore look for indices able to account for emergent behaviors (e.g. changes in plankton community and feeding behavior) triggered by increased nutrient loads. The trophic transfer efficiency (TTE), the efficiency at which carbon is transferred between trophic levels (Eddy *et al*., 2021), provides the conceptual basis to develop such an index. In the following sections, we propose a new eutrophication index and provide a preliminary assessment of its potential by using previously published biogeochemical simulations of the Baltic Sea, one of the most extensively studied basins in terms of eutrophication (Murray *et al*., 2019 and citation therein).

## THE TROPHIC TRANSFER INDEX

**Fig. 1:**
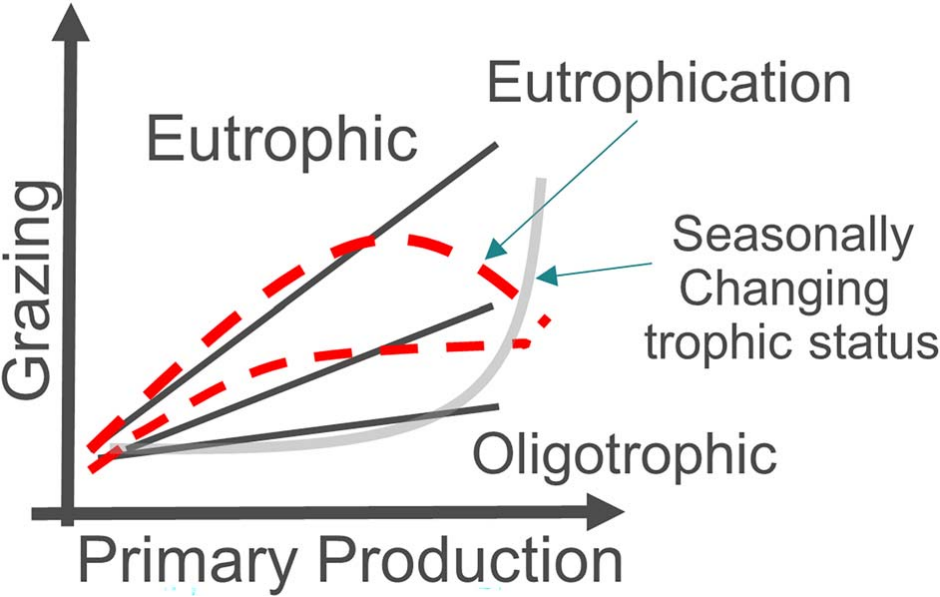
Schematic relationships between normalized PP and grazing. Each line is representative of a given ecosystem/site. Black continuous lines represent ecosystems with a constant TTE (TTE is given by the slope of the grazing vs. PP curve). Depending on the trophic conditions, the TTE changes with oligotrophic ecosystems generally displaying lower values (e.g. Armengol *et al*., 2019). According to our assumptions, these ecosystems are “healthy”. The gray line represents an ecosystem where TTE increases with PP (e.g. due to seasonally changing trophic conditions). In this case, the relationship between grazing and PP is higher than linear. According to our assumptions, this situation also implies healthy conditions. Finally, red dashed lines are representative of ecosystems/sites affected by eutrophication since TTE decreases for high PP values i.e. grazing does not monotonically increase with PP.

Our approach starts from the assumption that a specific site is affected by eutrophication if the amount of primary produced carbon that is channeled toward high trophic levels does not consistently increase with increasing PP (Tubay *et al*., 2013; Fig. 1, red lines). This assumption is supported by the evidence that all the events commonly associated with eutrophication are triggered by the prolonged presence of un-grazed PP in the water column (Chislock *et al*., 2013; EEA, 2019; Eddy *et al*., 2021).

**Fig. 2:**
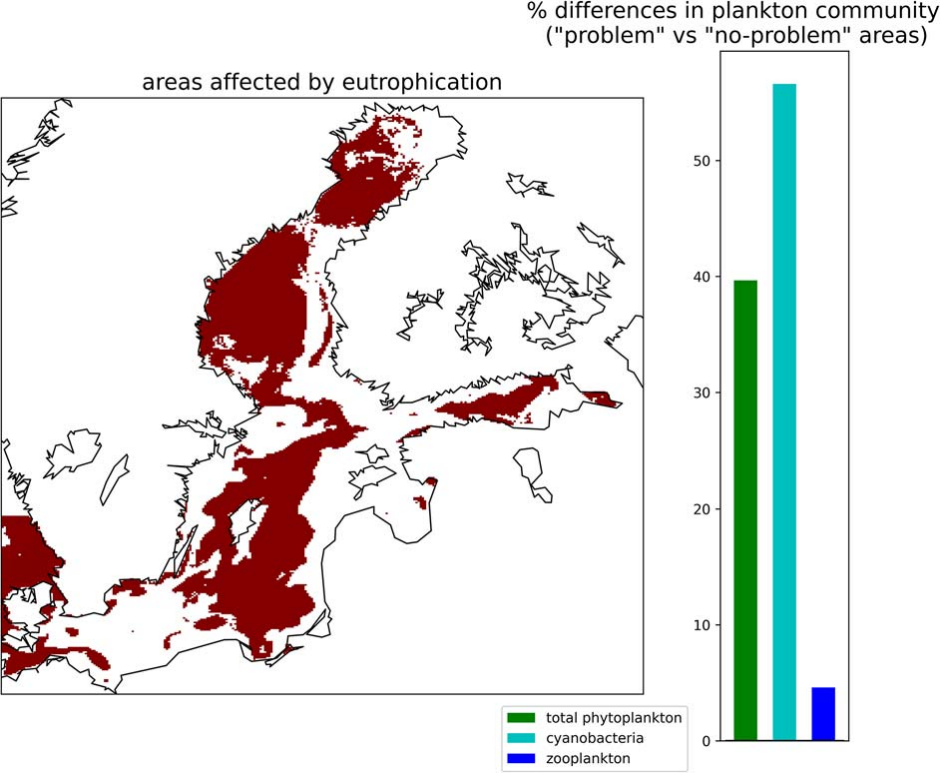
Simulated “problem areas” (left panel, dark red) and % differences in plankton community (right panel) between problem and no problem areas in the Baltic Sea. Percentages refer to spatially averaged and depth-integrated values of total phytoplankton, zooplankton and cyanobacteria biomass.

The main consequence of our assumption is that, in a healthy environment, and at appropriate time scales, grazing increases with PP following a linear or higher grade relationship (Kemp *et al*., 2001; Schmoker *et al*., 2013), regardless of the trophic status of the system (Fig. 1). The linearity of the PP versus grazing relationship can be assessed through the linear correlation coefficient (RL):


(1) \begin{equation*} RL= corrcoef\left( PP, Grazing\right) \end{equation*}

where $corrcoef$ is the function to calculate the Pearson correlation coefficient, and $PP$ and $Grazing$ (both given in mmol m^−2^ month^−1^) are the monthly, depth-integrated PP and grazing flux, respectively. Positive, higher grade relationships imply a monotonic increase in the slope of the PP versus grazing curve (Fig. 1, gray line), which can be assessed through the rank correlation coefficient (RR) between the grazing-to-PP ratio and PP:


(2) \begin{equation*} RR= Spearman\left( Grazing: PP, PP\right) \end{equation*}

where Spearman is the function to calculate the Spearman rank correlation coefficient. We then define the trophic transfer index as:


(3) \begin{equation*} {TT}_{ind}=\mathit{\max}\left( RL, RR\right) \end{equation*}

The condition to identify areas affected by eutrophication (hereafter “problem areas”, HELCOM, 2017; EEA, 2019) is that ${TT}_{ind}$ is below a specific threshold. We will discuss later possible criteria to choose this value. Here we highlight that, contrary to the traditional indicators based on biogeochemical concentrations, our approach implies only a single site-independent threshold.

We stress that in Equations (1–3) the use of PP and grazing is conceptually more robust than biomasses, as the latter do not allow an unambiguous discrimination between producers and consumers due to the widespread presence of mixoplankton within the marine protist community (Flynn *et al*., 2013; GonÇalves-Leles *et al*., 2018). The monthly time scale was chosen based on previous field (Widdicombe *et al*., 2010; Eloire *et al*., 2010) and modeling (Polimene *et al*., 2015) studies, suggesting that phytoplankton and zooplankton are linearly coupled at the monthly scale (i.e. the prey–predator cycle is shorter than 1 month). Depth-integration was performed to account for possible sub-surface maximum productivity (i.e. deep chlorophyll maximum layers).

## SIMULATING AREAS AFFECTED BY EUTROPHICATION: THE BALTIC SEA CASE STUDY

The trophic transfer index was tested in the Baltic Sea by using an established marine ecosystem model already implemented in that basin (Lessin *et al*., 2014; Pärn *et al*., 2020, https://ergom.net/). The index was computed in each grid point of the model domain by using the three yearlong simulations (2016–2018) presented in Macias *et al*. (2022). The fraction of problem areas identified was, as expected, dependent on the TVs used (Fig. S1). According to our assumption, a suitable TT-index threshold should be high enough to identify a healthy relationship between PP and grazing (i.e. high correlation between the two fluxes). We did not consider values <0.7, a threshold generally used in literature to mark high correlations between environmental variables (e.g. Serpetti *et al*., 2016; Coll *et al*., 2019). Furthermore, 0.7 was the value below which the significance of the correlations simulated by the model decreases sharply (Fig. S2). To test the reliability of TVs >0.7, we have applied the TT index in a known oligotrophic basin, the Mediterranean Sea (Karydis and Kitsiou, 2012). This exercise (Fig. S1) showed that for TVs >0.7, the percentage of the simulated problem areas would be unrealistically high for that basin (>30%), suggesting that TT index ≥0.7 is likely to imply healthy trophic interactions (i.e. not affected by eutrophication) between primary producers and consumers. For these reasons, we have selected 0.7 as the threshold for the preliminary assessment here presented.

Problem areas identified by the TT index (Fig. 2) cover 50% of the Baltic basin and include the southern part of the Bothnian Bay, the western part of the Bothnian Sea, the central part of the Gulf of Finland, most of the Gotland basin and the Kattegat. The areas classified as problem areas have on average more total phytoplankton biomass and cyanobacteria. Zooplankton biomass in these areas is higher than in the non-problem areas, but the difference is much lower than the difference in phytoplankton and cyanobacteria. Consequently, the zooplankton to phytoplankton ratio is lower.

**Fig. 3:**
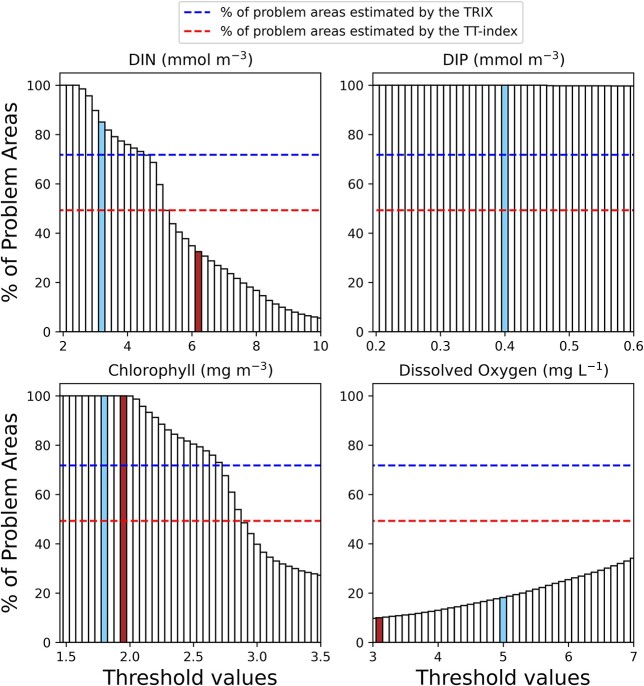
Percentage of problem areas in the Baltic Sea simulated by using traditional biogeochemical indicators as a function of the TVs, the TRIX (imposing TRIX>6) and the TT index. Colored bars correspond to TVs proposed by EU member states (A. Stips, Ispra, personal communication) for open sea (light blue) and coastal waters (brown). In the case of DIP, the two thresholds overlap.

Figure 3 shows the comparison between the fraction of the Baltic Sea classified as problem areas by the trophic transfer index and those classified by (simulated) traditional biogeochemical indicators (see SI for further details) as a function of the TVs used and the (simulated) TRIX index (Vollenweider *et al*., 1998; Fiori *et al*., 2016). The latter is calculated by combining four variables: total chlorophyll, dissolved inorganic nitrogen (DIN), dissolved inorganic phosphorus (DIP) and the absolute percentage deviation from O_2_ saturation (see SI for further details).

Biogeochemical indicators are often combined through the so-called one out all out principle (OOAO, EU Water Framework Directive, Borja and Rodríguez, 2010) implying that, when using multiple parameters to assess eutrophication, the final grade is based on the parameter that scores the worst. Applying this principle to the Baltic Sea, the eutrophication status of the basin is largely driven by the concentration of DIP. Considering TVs for DIP ≤0.6 μmol L^−1^ (this range does include the TV proposed by EU member states for open Seas and Coastal areas which is 0.4 μmol L^−1^), the whole basin is classified as a problem area regardless of the concentration of the other indicators. This result is in line with the assessment performed by the Baltic Marine Environmental Commission—HELCOM (HELCOM, 2017; EEA, 2019), stating that ca 96% of the Baltic Sea is affected by eutrophication. Interestingly, even when excluding DIP from the assessment and considering the TVs proposed by EU member states for chlorophyll (1.8 and 1.9 μg L^−1^ for open Seas and coastal waters, respectively; A. Stips, Ispra, personal communication) model simulations suggest that the whole basin is classifiable as a problem area. Only by considering DIN and bottom oxygen alone, the problem areas would be <100% of the basin (with variable percentages depending on the threshold used i.e. coastal vs. open waters). Also using the TRIX index, a large portion of the basin (72%) is classifiable as problem areas. On the contrary, if we consider the TT index, a considerably smaller part of the basin (ca. 50%) is classifiable as problem area. Therefore, the TT index underestimates the problem areas with respect to the classification made by traditional indicators combined through the OOAO principle, by DIP and chlorophyll considered alone and by the TRIX.

## SIGNIFICANCE, LIMITATIONS AND CONCLUDING REMARKS

The discrepancy between the TT index and the traditional indicators stems from the conceptual difference of the two approaches. Biogeochemical concentrations define the “status” of an ecosystem, whereas the TT index defines its “functionality”. We argue that we should base eutrophication assessments on the latter rather than the former. Indeed, a status characterized by high energy at the PP level (nutrients and light) resulting in high biomass is not a threat if that biomass is consistently channeled toward high trophic levels i.e. the functionality of the ecosystem is preserved (Nixon and Buckley, 2002; Eero *et al*., 2016). In the example here, circa 50% of the Baltic Sea, although displaying chlorophyll and phosphate concentrations higher than thresholds, does not present dysfunctional behaviors in the trophic interactions. As such, these areas should not be classified as problematic. We notice that the dysfunctional aspects identified by the TT index, a mismatch between phytoplankton and zooplankton and dominance of low palatable primary producers (in our example cyanobacteria, Pearl *et al*., 2001), are commonly associated with eutrophication (Cloern, 2001; Romero *et al*., 2013; Tubay *et al*., 2013; HELCOM, 2017); however, traditional indicators are unsuitable to detect them. For this reason, we propose that using biogeochemical concentrations as indicator could lead to misleading eutrophication assessments.

Although the mapping of eutrophication in the Baltic Sea identified by the TT index is partially consistent with what has previously been reported for this basin (HELCOM, 2017; EEA, 2019), the amount of problem areas is substantially less with respect to the assessment performed with the modeled traditional indicators. This result is preliminary and, at this stage, is not our intention to suggest the relaxation of existing targets and measures, especially considering the possible positive feedback of climate change to eutrophication dynamics (Rabalais *et al*., 2009). Nevertheless, we highlight the potential benefits the proposed approach might have on the management of the marine ecosystem, for example by avoiding unnecessarily strong measures triggered by an overestimation of the eutrophication risk.

We recognize that the application of the TT index in a real environmental context is problematic since PP and grazing are difficult to measure and not always included in monitoring programs. As shown in the example here, this limit can be overcome by using marine ecosystem models that offer numerical estimates of ecosystem functions not commonly measured in the field (e.g. grazing), with a spatial–temporal resolution that is impossible to achieve in reality. Nowadays models are routinely used for both scientific and management purposes (Piroddi *et al*., 2021b), and there is therefore the opportunity to create model-based eutrophication indices.

Making the TT index a fully model-based indicator inevitably adds the uncertainty of the simulated fluxes on which it is based (PP and grazing) to the eutrophication assessment. This aspect is especially relevant for the simulation of marine trophic fluxes, which are more complex and difficult to predict than their freshwater counterparts (Hessen and Kaartvedt, 2014). In particular, the largest source of uncertainty is currently associated with the simulations of grazing (Gentleman *et al*., 2003; Anderson *et al*., 2010; Chenillat *et al*., 2021), since most of the model developments has traditionally focussed on bottom-up mechanisms controlling phytoplankton growth (e.g. Litchman *et al*., 2007), with zooplankton-related dynamics receiving less attention (Mitra and Flynn, 2006; Sailley *et al*., 2015; Chenillat *et al*., 2021). Consequently, the capability of ecosystem models to simulate trophic interactions within the plankton community has only recently started to be assessed (Petrik *et al*., 2022). On the other hand, the potential of numerical tools for management purposes is increasingly recognized, as testified by the EU’s ambition to develop an Ocean Digital Twin (European Digital Twin of the Ocean, DTO) and efforts are in place to refine existing plankton models to make them closer to the real environment (Flynn *et al*., 2022). Our preliminary test suggests that plankton diversity is the key feature to include in models if we want to use numerical tools to assess, predict and prevent eutrophication. Although the model used here only accounts for a minimal description of biodiversity (3 phytoplankton functional types and only a single zooplankton), it clearly shows that the TT index is highly sensitive to the shift in the plankton community composition. We therefore expect that the index will be even more effective and informative when used in a modeling framework accounting for a more realistic description of functional diversity. The latter should be a priority in the development of DTOs. When such DTOs will be operative, the trophic transfer index could be routinely used to assess current and future eutrophication states of our seas. In the meantime, acknowledging that the reliability of the index is dependent on future model developments and validation, we propose it as a testing tool to complement more traditional, field-based, indicators and as a means to re-consider how eutrophication is currently defined, assessed and managed.

## Supplementary Material

Polimene_etal_2023_SI_revision_fbad022

## Data Availability

Data (simulations) used in this paper are available on request.
