# Supplementary material for: Should we reconsider how to assess eutrophication?
Source: J Plankton Res. 2023 May 25;45(3):413–20. doi: 10.1093/plankt/fbad022 (PMC10243849; doi:10.1093/plankt/fbad022)
Supplement: Polimene_etal_2023_SI_revision_fbad022 [file polimene_etal_2023_si_revision_fbad022.docx]

**Supplementary Information (SI)**

**Simulated biogeochemical indicators and TRIX index**

For the analysis of the biogeochemical indicators (dissolved inorganic nitrogen and phosphorus, chlorophyll and bottom oxygen), we have considered, for nutrients, the averages of the surface values simulated in winter months (Dec-Feb) and, for chlorophyll, the averages of the surface values simulated in summer (Jun-Sept, Stips et al. 2016). For bottom oxygen, we used the minima monthly values of the whole simulation. Eutrophication ratios (ER=simulated values/ TVs, HELCOM, 2017), calculated in each grid point of the model domain, have been used to identify problem areas (ER>1). TV ranges have been chosen to be wide so to include both coastal and open sea values.

The TRIX index (Vollenweider et al., 1998; Fiori et al., 2016; Stips et al., 2016) is calculated by combining 4 variables $\left( n \right)$: total chlorophyll (Chl, mg m^-3^), dissolved inorganic nitrogen (DIN, mmol m^-3^) dissolved inorganic phosphorus (DIP, mmol m^-3^) and the absolute percentage deviation from O_2_ saturation (DO=$\left| 100-O_{sat} \right|$). Each of these variables ($M_{i}=Chl,DIN,DIP, DO)$ is scaled by a maximum ($U_{i}$) and a minimum ($L_{i}$) value (Table S1):

$$TRIX=\frac{10}{n}\sum_{i=1}^{n} \frac{\left( {logM}_{i}-{logL}_{i} \right)}{\left( {logU}_{i}-{logL}_{i} \right)}$$

**Table S1**. Minimum (U) and maximum (L) values used for the simulated TRIX (Stips et al., 2016)

|  | Max (U) | Min (L) |
| --- | --- | --- |
| Chl (mg m^-3^) | 60 | 0.01 |
| DIN (mmol m^-3^) | 60 | 0.001 |
| DIP (mmol m^-3^) | 10 | 0.001 |
| DO (%) | 100 | 0.1 |

The TRIX was computed in each grid point of the model domain and averaged over the whole simulation. The percentage of problem areas was identified by imposing TRIX > 6 (Fiori et al., 2016)


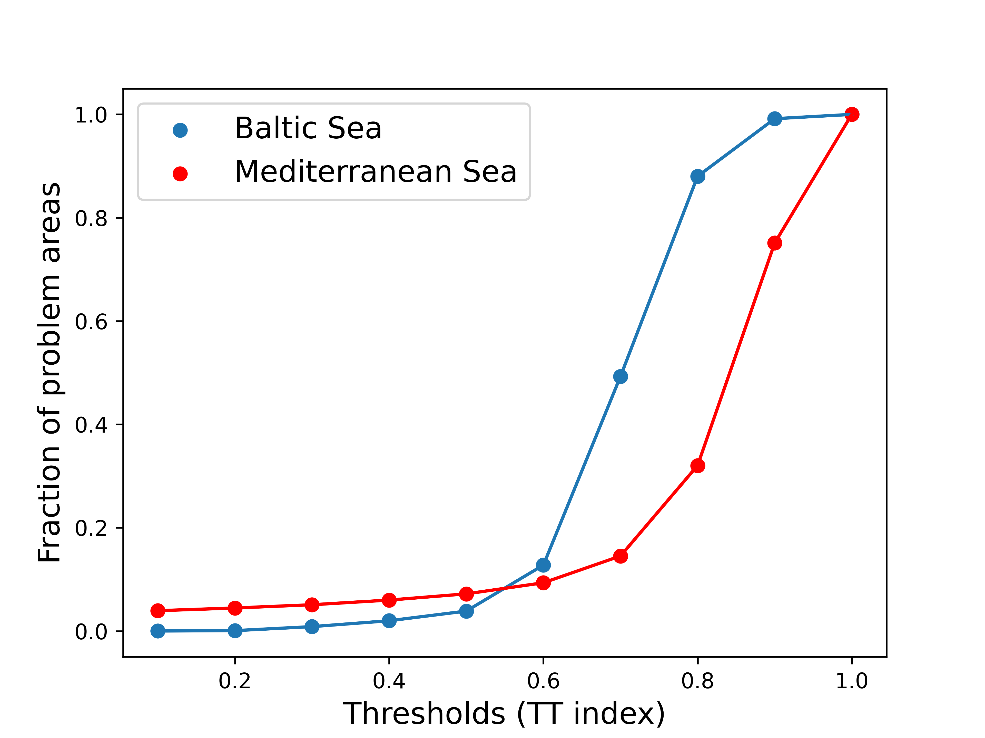


**Fig S1**. Fraction of simulated problem areas as function of the trophic transfer index thresholds in the Baltic and the Mediterranean Seas


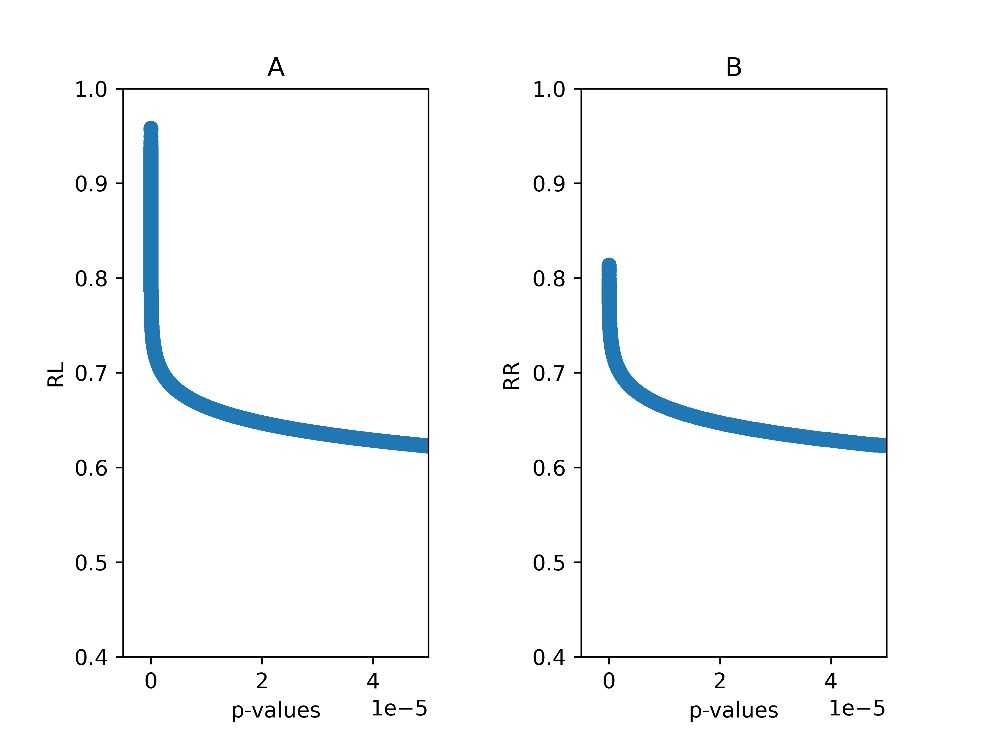


**Fig S2**. Linear (A) and rank (B) correlation coefficients (RL and RR, respectively) as function of the p-values in the Baltic Sea

**References**

Fiori, E., Zavatarelli, M., Pinardi, N., Mazziotti, C., Ferrari, C. R. (2016). Observed and simulated trophic index (TRIX) values for the Adriatic Sea basin. Nat. Hazards Earth Syst. Sci., 16: 2043–2054, https://doi.org/10.5194/nhess-16-2043-2016, 2016.

HELCOM, (2017). The integrated assessment of eutrophication — supplementary report to the first version of the 'State of the Baltic Sea' report. 2017. Baltic Marine Environment Protection Commission, Helsinki, Finland. Available at: <http://stateofthebalticsea.helcom.fi/about-helcom-and-the-assessment/downloads-and-data/>

Stips, A., Macias, D., Garcia-Gorriz, E., Miladinova, S. (2016). Alternative assessments of large scale Eutrophication using ecosystem simulations: hind-casting and scenario modelling; EUR 27904; doi:10.2788/156650

Vollenweider, R.A., Giovanardi, F., Montanari, G., Rinaldi, A., (1998) Characterization of the trophic conditions of marine coastal waters with special reference to the NW Adriatic Sea: proposal for a trophic scale, turbidity and generalized water quality index. Environmetrics, 9: 329-357.
